# Establishment and optimization of a hemp (*Cannabis sativa* L.) agroinfiltration system for gene expression and silencing studies

**DOI:** 10.1038/s41598-020-60323-9

**Published:** 2020-02-26

**Authors:** Michihito Deguchi, Daniel Bogush, Hannah Weeden, Zachary Spuhler, Shobha Potlakayala, Takumasa Kondo, Zhanyuan J. Zhang, Sairam Rudrabhatla

**Affiliations:** 1 Penn State Harrisburg, 777 West Harrisburg Pike, Middletown, Pennsylvania, USA; 2 AGROSAVIA, Centro de Investigación Palmira, Calle 23, Carrera 37, Continuo al Penal Palmira, Valle, Colombia; 3 Plant Biotechnology Innovation Laboratory, Division of Plant Sciences, University of Missouri, Columbia, Missouri USA

**Keywords:** Plant biotechnology, Plant molecular biology

## Abstract

Industrial hemp (*Cannabis sativa* L.) is a high-yielding annual crop primarily grown for fiber, seeds, and oil. Due to the phytochemical composition of hemp, there has been an increased interest in the market for nutraceuticals and dietary supplements for human health. Recent omics analysis has led to the elucidation of hemp candidate genes involved in the syntheses of specialized metabolites. However, a detailed study of these genes has not been undertaken due to the lack of a stable transformation system. We report for the first time an agroinfiltration system in hemp utilizing vacuum infiltration, which is an alternative method to stable transformation. A combination of 0.015% Silwett L-77, 5 mM ascorbic acid, and thirty second sonication followed by a 10-minute vacuum treatment resulted in the highest β-glucuronidase expression in the leaf, male and female flowers, stem, and root tissues. The phytoene desaturase gene was silenced with a transient hairpin RNA expression, resulting in an albino phenotype in the leaves and the male and female flowers. This agroinfiltration system would be useful for overexpression and silencing studies of target genes to regulate the yield of specialized metabolites in hemp.

## Introduction

Industrial hemp (*Cannabis. sativa* L.) is a diploid (2n = 20) and typically dioecious plant primarily grown for fiber, seeds, and oil. Recently, this crop has been exploited for medicinal compounds as it produces 565 secondary metabolites, such as cannabinoids, terpenes and other phenolic compounds^1^. Unlike drug-type *Cannabis* (marijuana) that produces high levels of the psychoactive component Δ9-tetrahydrocannabinol (THC), hemp preferentially produces cannabidiol (CBD) and low amounts of THC (0.3% compared with marijuana). In addition, a total of 25,000 hemp products are currently produced and sold on the market, for both scientific and industrial purposes^2^.

Cannabinoids are terpenophenolic compounds that include more than 100 chemical compounds, such as THC and CBD^2^. These molecules have pharmacological properties in the treatment of pain, mood disorders, diabetes, cancer, and neurodegenerative and inflammatory diseases^3,4^. The biosynthesis of these molecules occurs in the glandular trichomes located predominantly on the flowers and leaves of *C. sativa*^1^. The cannabinoid pathway is initiated by the synthesis of olivetolic acid (OA) originating from the primary metabolite precursor via hexanoyl-CoA by tetraketide synthase (type III polyketide synthase) and olivetolic acid cyclase^5^. Together with geranyldiphosphate (GPP) from the plastidal 2-C- methyl-D-erythritol-4-phosphate pathway, OA is converted into cannabigerolic acid (CBGA) by CBGA synthase. This cannabinoid pathway concludes with the syntheses of tetrahydrocannabinolic acid (THCA) and cannabidiolic acid (CBDA) by THCA synthase (THCAS) and CBDA synthase (CBDAS), respectively^6^. THCAS and CBDAS are converted to THC and CBD, respectively, by nonenzymatic reactions.

Terpenes have anxiolytic, antibacterial, anti-inflammatory, and sedative effects on human diseases^7^. For example, the sesquiterpene β-caryophyllene interacts with mammalian cannabinoid receptors to reduce inflammation^8^. The terpene biosynthesis pathway in *C. sativa* includes two pathways: the plastidial methylerythritol phosphate (MEP) pathways, which leads to the production of monoterpenes, and the cytosolic mevalonate (MEV) pathway, which leads to the production of sesquiterpenes^1^. Seven enzymes have been shown to produce two terpene precursors: GPP and farnesyl diphosphate (FPP). Furthermore, the nine terpene synthase genes that catalyze terpene production from GPP and FPP have been identified^9^.

Knowledge of these secondary metabolite synthesis genes will help facilitate genetic improvements to obtain desirable metabolite profiles. However, these candidate genes were proposed based on genomic, transcriptomic and proteomic analyses and remain to be studied. Towards this goal, stable transformation is ideal; however, a hemp transformation protocol has not yet been developed due to the low shoot regeneration rate^10,11^.

Agroinfiltration is a prominent methodology for transiently expressing or silencing genes of interest in an efficient manner. This method can be used to produce vaccines and enzymes for industrial use as an alternative to stable transformation^12^. Agroinfiltration studies have also provided insight into various biological processes, such as promoter function, gene expression, subcellular protein localization, protein-protein interactions, and metabolism^13^. To date, this agroinfiltration system has been developed and utilized in both 1) model plant species such as tobacco, *Nicotiana benthamiana*, *Arabidopsis*, tomato and 2) agronomic crops such as rice, soybean, onion, flax, cowpea, grapevine, rose, and cacao^14^. Depending on the plant species, the agroinfiltration efficiency varies, resulting in high expression in plants such as *N. benthamiana* and tobacco or low expression in a wide range of plant species, including hemp. For infection of the plant leaf cells, *Agrobacterium* suspension is injected into the leaf parenchyma, but in many cases, agroinfiltration is not successful because the morphology of the leaf and particularly the structure of the thick epidermis do not allow the bacterial culture to enter the parenchyma, consequently resulting in a low gene expression efficiency^15^.

In hemp, a hairy root culture system for transient gene expression assays was developed and resulted in slight β-glucuronidase (GUS) positive staining using *Agrobacterium tumefaciens* and *A. rhizogenes*^16^. However, pharmaceutical components are not abundant in the *C. sativa* root^17^, and therefore, this system is not appropriate for analyses of the genes encoding enzymes that synthesize specialized metabolites.

To our knowledge, this article constitutes the first report of *C. sativa* agroinfiltration with a high efficacy in the aerial parts of the plant where pharmaceutical components are highly synthesized, transported and accumulated. The agroinfiltration method was optimized by a combination of physical and chemical treatments with different *Agrobacterium* strains and hemp cultivars. This optimized system had a high efficiency for exogeneous gene expression and silencing of endogenous genes in different hemp tissue/organs.

## Results

### Optimization of agroinfiltration

**Figure 1:**
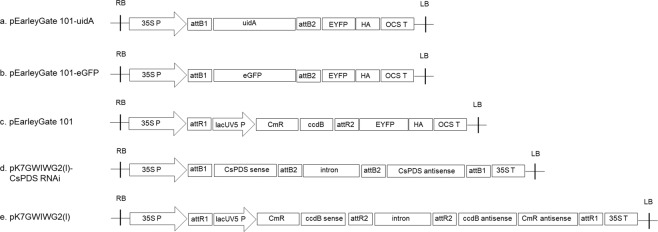
Schematic of the T-DNA structure of the binary vectors used for transient gene expression. (**a**) *uidA* gene (ORF is 1806 bp); (**b**) *eGFP* gene (ORF is 723 bp); (**c**) pEarleyGate 101; (**d**) Sense and antisense partial *PDS* fragment (601–800 bp); (**e**) Empty pK7GWIWG2(I) as the negative control; 35 S P: Cauliflower mosaic virus 35 S promoter; lacUV5P: lacUV5 promoter; OCS T: Terminator of the octopine synthase gene; 35 S T: Cauliflower mosaic virus 35 S promoter; attB1 and attB2: Gateway BP reaction recombination site; attRI and attR2: Gateway LR reaction recombination site; ccd8: *E. coli* toxic protein coding gene; CmR: Chloramphenicol resistance gene; RB: Right border; LB: Left border.

The *GUS* gene (*uidA*), which encodes the enzyme GUS, is one of the most widely used reporter genes in plant molecular biology^18^. For analysis of the efficiency of agroinfiltration, the *uidA* gene was cloned into the binary vector pEarleyGate 101 and expressed under the control of the cauliflower mosaic virus (CaMV) 35 S promoter (Fig. 1a). This cassette was transformed into *Agrobacterium* GV3101. Further agroinfiltration was carried out, and the tissues underwent histochemical GUS staining.

Agroinfiltration was carried out via vacuum infiltration to test the different tissues of the plant. The mature leaf, male flower, female flower, stem and root tissues were excised from the hemp plants two months after seed germination. In a histochemical GUS assay, only the male flower in all the tissues tested showed weak GUS staining. Then, various parameters such as chemical additives, sonication, different vacuum time, hemp cultivars and *Agrobacterium* strains were optimized using the male flower to create an optimal agroinfiltration protocol.

### Effect of chemical additives

The effect of four chemical additives including Silwett L-77, Pluronic F-68, L-Ascorbic acid and polyvinylpyrrolidone (PVP) was evaluated at different concentrations.

**Figure 2:**
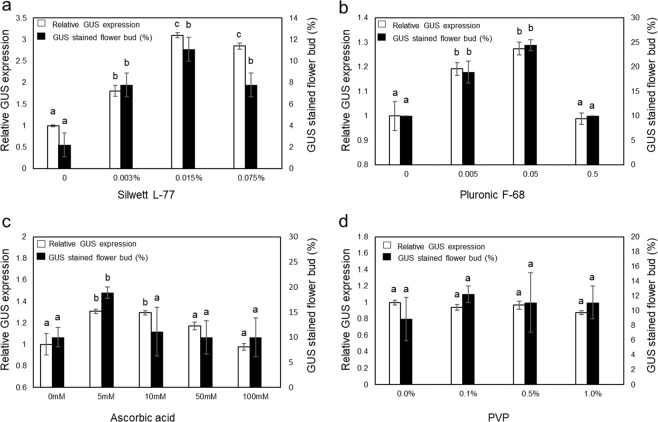
Effect of chemical additives on transient GUS expression. *Agrobacterium* GV3101 harboring pEarleyGate 101-*uidA* was infiltrated with different chemical additives; (**a**) Silwett L-77, (**b**) Pluronic F-68, (**c**) ascorbic acid and d) PVP as measured by image analysis of the GUS-stained female flowers collected 45 days after germination. The total GUS expression value without additives in each figure was used to normalize the relative expression level. Bars indicate ± standard error of the mean. Means followed by the same letter indicate no significant difference (P < 0.05). Statistical analysis was completed using a 1-way ANOVA with Tukey’s multiple comparison test.

Silwett L-77 and Pluronic F-68 are surfactants that can reduce the surface tension and enhance the entry of bacteria into plant tissues^19^. The addition of Silwett L-77 at 0.015% drastically increased the GUS expression to approximately three times that of the negative control plant (0% Silwett L-77) (Fig. 2a). The percentage of the GUS-stained male flowers was higher in the 0.015% Silwett L-77 group (11.1%) than the negative control group (2.2%). This effect was significant for both relative GUS expression and the percentage of GUS-stained female flowers with 0.015% Silwett L-77. Therefore, Silwett L-77 at a final concentration of 0.015% was added to the agroinfiltration media for all subsequent agroinfiltration experiments. Addition of Pluronic F-68 led to a 25% increase in the relative GUS expression and a 2.5-fold increase in the percentage of GUS-stained female flowers (24.4%) at a concentration of 0.05%, while 0.5% did not display any positive effect (Fig. 2b), presumably due to tissue damage by the high concentration of this surfactant (Fig. 2b).

Ascorbic acid and PVP were tested because of their roles as antioxidants that scavenge the excess reactive oxygen species (ROS) produced by *Agrobacterium* infection. Ascorbic acid concentrations correlated with increased relative GUS expression (1.3-fold increase compared to the negative control) and a higher percentage of GUS-stained female flowers (18.9%) compared with the negative control (10%), with a final concentration of 5 mM (Fig. 2c). No significant increases in the GUS level were observed in the plants treated with PVP (Fig. 2d).

### Vacuum and sonication time

**Figure 3:**
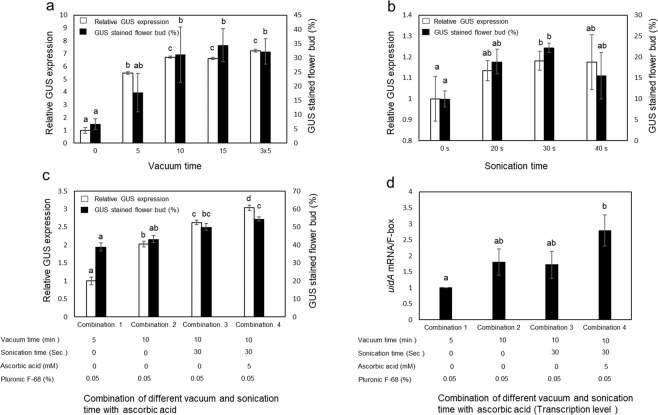
Effect of various treatments on the transient GUS expression. Effect of (**a**) Vacuum time; (**b**) Sonication time; (**c**) Combination of different vacuum times, sonication times and ascorbic acid concentrations. (**d**) Transcriptomic analysis of the combination of different vacuum times, sonication times and different concentrations of ascorbic acid. The control conditions were 5 minutes of vacuum, no sonication and no ascorbic acid to normalize the *GUS* gene expression. Bars indicate ±standard error of the mean. Identical letters indicate no significant difference (P < 0.05). Statistical analysis was performed using a 1-way ANOVA with Tukey’s multiple comparison test.

Vacuum infiltration for 5 minutes resulted in a higher relative GUS expression (5.5-fold) and percentage of GUS-stained flower buds (17.7%) than the non-vacuum treatment (Fig. 3a). Increasing the vacuum time to 10 minutes and 15 minutes and three iterations of 5 minutes resulted in a higher relative GUS expression than that with 5 minutes of vacuum treatment, but there was no significant effect on the percentage of GUS-stained flower buds (Fig. 3a).

Exposure of the plant to sonication in the presence of *Agrobacterium* produces many microwounds across the tissue, which allows the *Agrobacterium* to penetrate deeper and more completely throughout the tissue^15^. Sonication for 30 seconds before vacuum infiltration had a significant effect on the nonsonicated male flowers for both relative GUS expression (1.2-fold) and the percentage of GUS-stained male flowers (22.2%; Fig. 3b). A combination of different vacuum and sonication times with ascorbic acid was tested. Ten minutes of vacuum infiltration after 30 seconds of sonication together with the addition of 0.05% Pluronic F-68 and 5 mM of ascorbic acid resulted in GUS staining of more than 50% of the male flowers and the highest relative GUS expression (3-fold) and *GUS* transcript level (Fig. 3c,d).

### *Agrobacterium* strain and hemp cultivars

**Figure 4:**
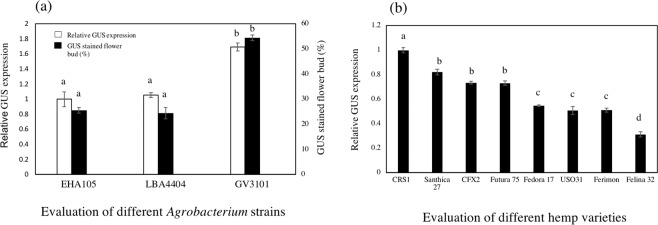
Effect of the *Agrobacterium* strains on the transient GUS expression in male flowers (**a**). Effect of the hemp cultivars on the transient GUS expression in the mature leaf (**b**). Bars represent ± standard error of the mean. Identical letters indicate no significant difference (P < 0.05). Statistical analysis was performed using a 1-way ANOVA with Tukey’s multiple comparison test.

Three disarmed *Agrobacterium* strains, EHA105, LBA4404 and GV3101 containing the pEarlyGate 101-*uidA* binary vector, were used to perform the histochemical GUS assays. Among the three *Agrobacterium* strains, GV3101 demonstrated an approximately 1.7-fold increase in GUS expression compared to the other two strains (Fig. 4a); therefore, GV3101 was used to evaluate the effect of hemp cultivars on the transient GUS expression.

Since the structures of the male flowers are different between monoecious and dioecious cultivars, it is not possible to compare the transient GUS expression in male flowers. Therefore, mature leaves were used to evaluate the effect of the hemp cultivars on the transient GUS expression. The dioecious CRS-1 cultivar had the highest relative GUS expression (Fig. 4b). Santhica 27, CFX-2 and Futura 75 exhibited approximately 80% of the activity compared to CRS-1. Fedora17, USO31 and Ferimon showed approximately 60% relative GUS expression compared to CRS-1. Felina 32 showed the lowest GUS expression among all eight cultivars tested (Fig. 4b).

### Transient gene expression in diverse hemp tissues/organs

**Figure 5:**
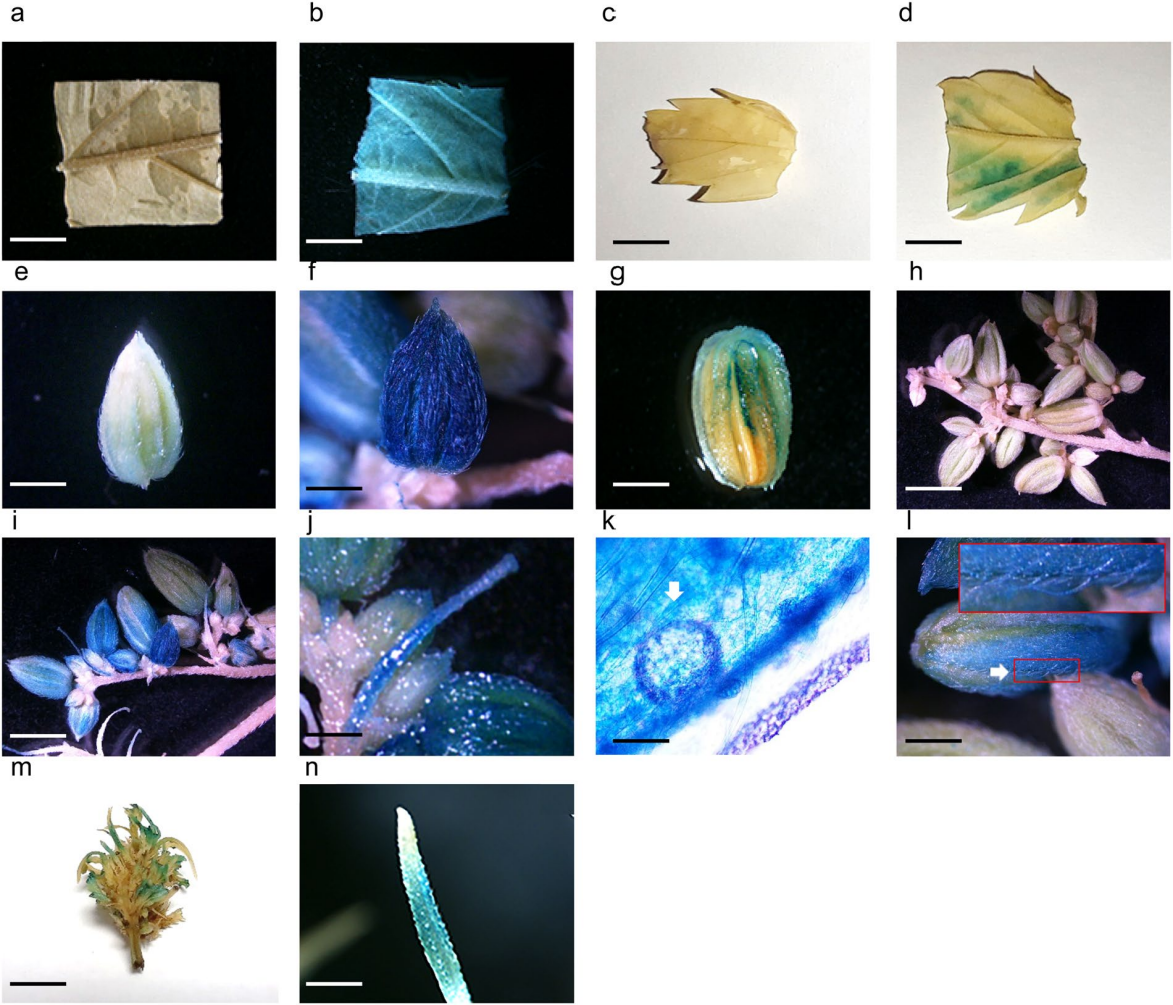
GUS staining in various CRS-1 tissues/organs. The pEarleyGate 101-*uidA* transformed *Agrobacterium* GV3101 culture that contains 0.015% of Silwett L-77, 0.05% of Pluronic F-68 and 5 mM of ascorbic acid was vacuum infiltrated for 10 minutes after 30 seconds of sonication. The pictures of GUS stained tissues/organs were taken 4 days after infiltration. (**a**) Control hemp leaf discs (bar 500 µm). (**b**) Agroinfiltrated leaf discs (bar 500 µm). (**c**) Control mature leaf (bar 15 mm). (**d**) Agroinfiltrated mature leaf (bar 15 mm). (**e**) Control pollen sac (bar 600 µm). (**f**) Agroinfiltrated pollen sac (bar 600 µm). (**g**) Agroinfiltrated anthers and sepals (bar 120 µm). (**h**) Control pollen sac clusters (bar 200 µm). (**i**) Agroinfiltrated pollen sac clusters (bar 200 µm). (**j**) Agroinfiltrated filaments (bar 120 µm). (**k**) Agroinfiltrated pollen (bar 25 µm). (**l**) Agroinfiltrated nonglandular trichomes (bar 400 µm). (**m**) Agroinfiltrated female flower (bar 13 mm). (**n**) Agroinfiltrated pistil (bar 300 µm). Arrows indicate pollen grain and nonglandular trichomes in (**k**,**l**), respectively.

The optimized hemp agroinfiltration protocol was applied to a wide range of hemp tissues/organs. Successful GUS staining was observed in the mature leaf discs (Fig. 5b), mature leaves (Fig. 5d), pollen sacs (Fig. 5f), anthers and sepals (Fig. 5g), pollen sac clusters (Fig. 5i), filaments (Fig. 5j), pollen grains (Fig. 5k), nonglandular trichomes (Fig. 5l), female flowers (Fig. 5m) and pistils (Fig. 5n). The empty pEarleyGate 101 vector did not result in GUS staining in the mature leaf discs (Fig. 5a), mature leaves (Fig. 5c), pollen sacs (Fig. 5e) and pollen sac clusters (Fig. 5h).

**Figure 6:**
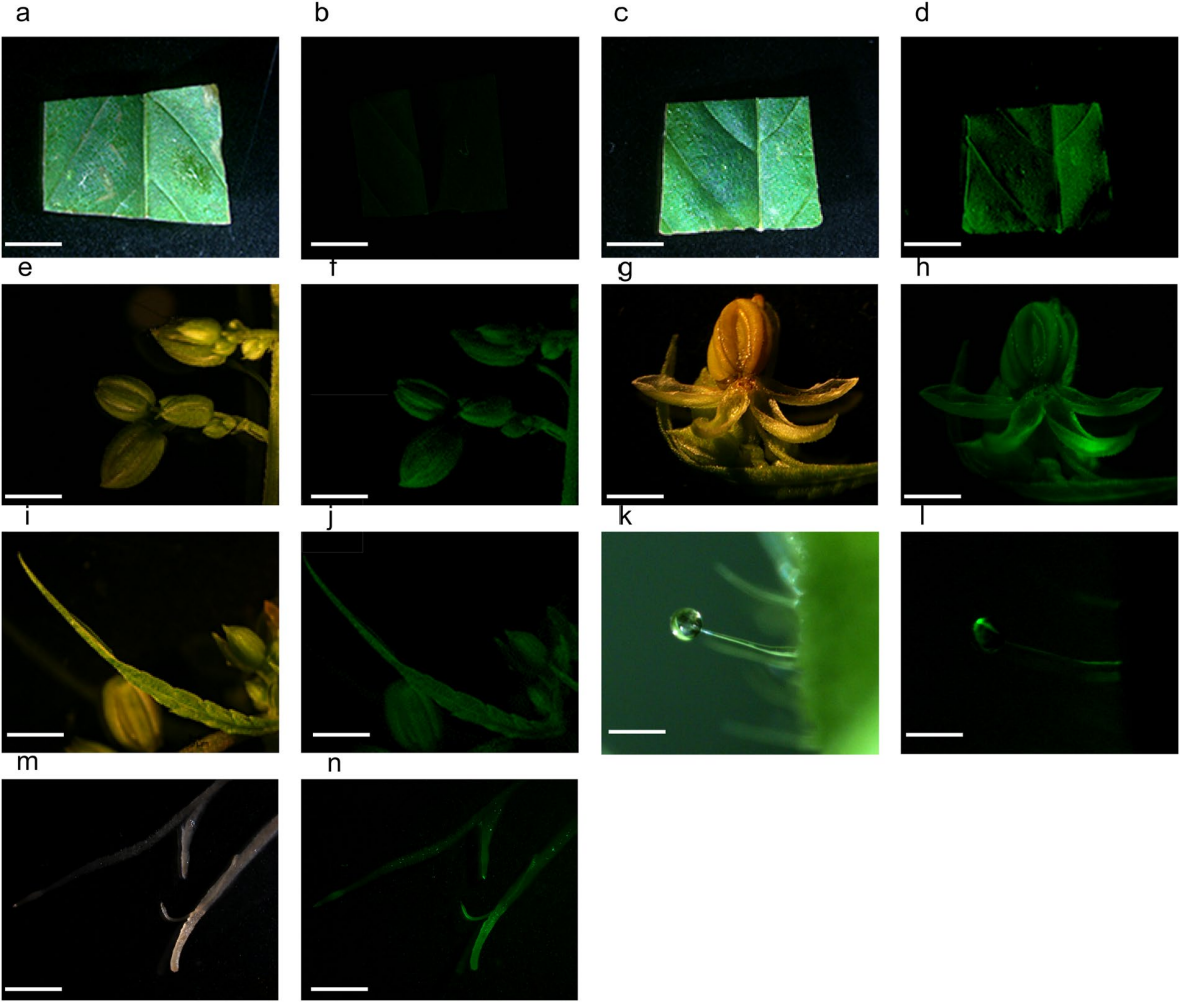
Transient GFP expression in various CRS-1 tissues/organs. The pEarleyGate 101-eGFP transformed *Agrobacterium* GV3101 culture that contains 0.015% of Silwett L-77, 0.05% of Pluronic F-68 and 5 mM of ascorbic acid was vacuum infiltrated for 10 minutes after 30 seconds of sonication. The pictures of GFP fluorescence were taken 4 days after infiltration. (**a**). Bright field image of an empty vector pEarleyGate 101-treated leaf disc. Bar 800 µm (**b**). GFP fluorescence of an empty vector pEarleyGate 101-treated leaf disc. Bar 800 µm (**c**). Bright field image of a pEarleyGate 101-*eGFP*-treated leaf disc. Bar 800 µm (**d**). GFP fluorescence of a pEarleyGate 101-*eGFP*-treated leaf disc (**e**). Bright field image of a pEarleyGate 101-eGFP-treated pollen sac cluster. Bar 200 µm. (**f**). GFP fluorescence of a pEarley Gate 101-*eGFP*-treated pollen sac cluster. Bar 200 µm. (**g**). Bright field image of pEarleyGate 101-*eGFP*-treated anther and sepal. Bar 120 µm (**h**). GFP fluorescence of pEarley Gate 101-*eGFP*-treated anthers and sepals. Bar 120 µm (**i**). Bright field image of a pEarleyGate 101-*eGFP*-treated filament. Bar 200 µm (**j**). GFP fluorescence of a pEarley Gate 101-*eGFP*-treated filament. Bar 200 µm (**k**). Bright field image of a pEarleyGate 101-*eGFP*-treated capitate-stalked trichome. Bar 150 µm (**l**). GFP fluorescence of a pEarley Gate 101-*eGFP*-treated capitate-stalked trichome. Bar 150 µm (**m**). Bright field image of a pEarleyGate 101-*eGFP*-treated root from a hemp seedling. Bar 10 mm (**n**). GFP fluorescence of a pEarley Gate 101-*eGFP*-treated root from a hemp seedling. Bar 10 mm.

The pEarleyGate 101-*eGFP* vector (Fig. 1b) was also transiently expressed via a GV3101 strain of *Agrobacterium*. GFP fluorescence was detected in the hemp leaf discs (Fig. 6d), pollen sac clusters (Fig. 6f), anthers and sepals (Fig. 6h), filaments (Fig. 6j), capitate-stalked trichomes (Fig. 6l) and roots in the hemp seedlings (Fig. 6n). The hemp leaf discs filtrated by the empty pEarley vector did not show any GFP fluorescence (Fig. 6b). The bright field image of a leaf disc that was treated with the empty vector pEarleyGate 101 is shown in Fig. 6a. The bright field images of the leaf discs, pollen sacs, anthers and sepals, filaments, capitate-stalked trichomes and roots that were treated with pEarleyGate 101-*eGFP* are shown in Fig. 6c,e,g,i,k,m, respectively.

### Silencing of the phytoene desaturase (PDS) gene by an RNAi vector

**Figure 7:**
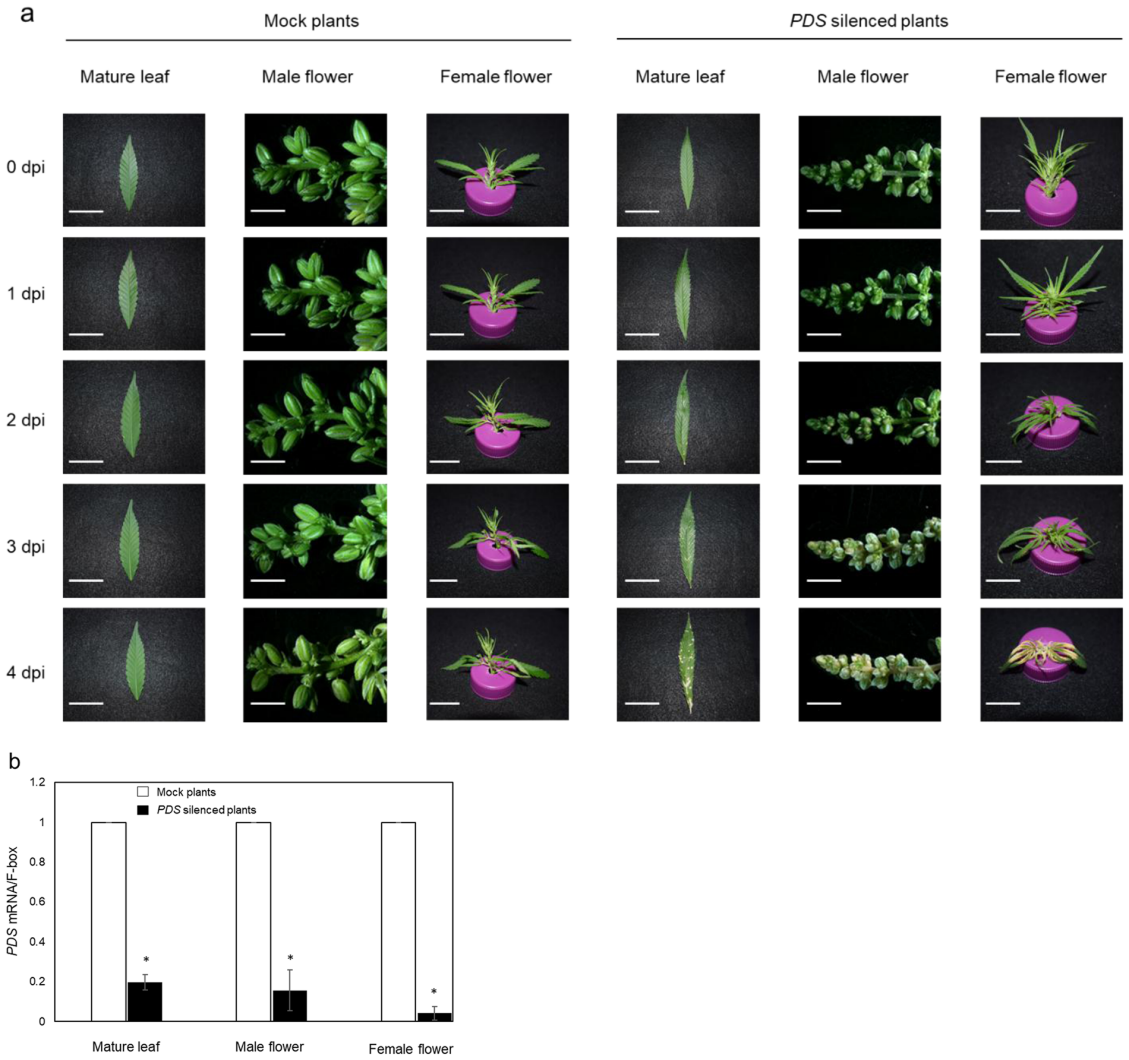
Silencing of hemp PDS gene expression by pK7GWIWG2(I)-*CsPDS* RNAi. (**a**) Phenotype of hemp mature leaf (bar 15 mm), male flower (bar 5 mm) and female flower (bar 10 mm) caused by silencing of the PDS gene. The pictures were taken at 0, 1, 2, 3 and 4 days postinfection (dpi). Ten leaves, ten male flowers and ten female flowers were agroinfiltrated and seven leaves, eight male flowers and eight female flowers showed the albino phenotype. (**b**) Analysis of the PDS gene expression to study the efficiency of gene silencing by agroinfiltration. The empty vector pK7GWIWG2(I) was transiently expressed (Mock) and used for normalization of PDS gene expression. Bars ± standard error of the mean. Asterisk indicates a significant difference in the statistical analysis (P < 0.05) in paired t-tests.

We chose the CsPDS gene that encodes the phytoene desaturase for *Agrobacterium*-mediated gene silencing because of its visible mutant phenotype caused by the lack of carotenoids^20,21^. The 200 bp fragment of the hemp PDS gene (accession no. PK24508.1, nucleotide no. 601–800) was amplified from the hemp leaf cDNA library and used to hairpin-based RNAi vectors. The *PDS* silencing vector was designed to include both sense and antisense strands of the *CsPDS* fragment to form the hairpin RNA. This silencing vector was transformed into the GV3101 strain and used for hemp agroinfiltration. Four days after inoculation, an albino phenotype was observed in the mature leaves, male flowers and female flowers (Fig. 7a). qPCR analysis demonstrated that the CsPDS gene expression was sharply decreased to less than 20% compared with that of the control tissues that were inoculated by GV3101 harboring the empty vector pK7GWIWG2(I) (Fig. 7b).

## Discussion

Vacuum agroinfiltration was attempted in mature leaves, male flowers, and female flowers, but only the male flowers exhibited a low transient GUS expression. Then, we used male flowers to optimize the hemp agroinfiltration. A concentration of 0.015% Silwett L-77 drastically increased the transient GUS expression and resulted in three times higher relative GUS expression than that without Silwett L-77, demonstrating that Silwett L-77 is a critical factor for successful hemp vacuum agroinfiltration (Fig. 2a). Thus, this concentration was used for all subsequent agroinfiltration experiments. Pluronic F-68 at 0.05% also increased the GUS activity by approximately 20% (Fig. 2b). The efficiency of both surfactants on the GUS expression in the hemp male flower (Fig. 2a,b) proved that the addition of these surfactants effectively increased the agroinfiltration efficiency in hemp as well as various crops^22,23^.

In plant cells, agroinfiltration triggers an oxidative burst response and induces the accumulation of ROS that injures plant cells and leads to necrosis^24^. Excess levels of ROS were reported to reduce the ability for *Agrobacterium* to colonize plant cells and transfer T-DNA into the nuclei^25^. For scavenging of the excess ROS, two antioxidant compounds were tested: ascorbic acid and PVP^26,27^. A 5 mM concentration of ascorbic acid had a significant and positive effect on transient GUS activity and increased the relative GUS expression by 50% (Fig. 2c), whereas PVP did not significantly improve the efficiency of agroinfiltration (Fig. 2d). These results are consistent with a *N. benthamiana* infiltration study previously reported by Norkunas *et al*. (2018)^22^.

For vacuum agroinfiltration, plant tissue was submerged into the suspension of *Agrobacterium* harboring a binary vector. Application of a vacuum results in evacuation of the air in the interstitial space from the submerged plant tissue through the stomata. The suspension of *Agrobacterium* enters the plant tissue to replace the evacuated gases once the vacuum is broken and the internal pressure is increased. Long periods of vacuum time lead to damaged plant tissues and short vacuum times result in insufficient air evacuation and low agroinfiltration efficiency^14^. In this study, both 10 minutes and 15 minutes as well as 3 iterations of 5 minutes of the vacuum treatment resulted in approximately seven times higher relative GUS expression than that of the control (non-vacuum) male flower. In statistical tests, these values were significantly higher than those after shorter periods of vacuum application for both relative GUS expression and the percentage of GUS-stained flower buds (Fig. 3a).

Sonication leads to the formation of microwounds within the plant tissue, allowing *Agrobacterium* to effectively infect the plant cells^28^. Since this treatment produces more entry points for *Agrobacterium* to enter into the plant cell, sonication facilitates agroinfiltration in recalcitrant plants. We performed tests to determine the best sonication time for hemp and found that 30 seconds of sonication before vacuum infiltration led to 20% higher relative GUS expression than that without sonication (Fig. 3b).

Thus, Silwett L-77, Pluronic F-68, ascorbic acid, vacuum and sonication all proved to be effective for hemp agroinfiltration. To further optimize the agroinfiltration efficiency, we tested a combination of Silwett L-77, Pluronic F-68, ascorbic acid, vacuum and sonication. As a consequence, ten minutes of vacuum infiltration after 30 seconds of sonication together with the addition of 0.015% Silwett L-77, 0.05% Pluronic F-68 and 5 mM of ascorbic acid resulted in 2.5-fold higher *uidA* gene expression and staining of more than 50% of the male flowers than those of the Silwett L-77 only group (Fig. 3c). This result indicates the synergistic effect of Silwett L-77, Pluronic F-68, ascorbic acid and acetosyringone.

The genetic background of the *Agrobacterium* can influence the T-DNA transfer into plant cellular nuclei, and some *Agrobacterium* strains are more virulent than others based on the target species^29,30^. In this study, different transformation rates were observed for three avirulent strains. To the best of our knowledge, there are few reports about appropriate *Agrobacterium* strains for hemp due to the limited tissue culture and transformation studies. The GV3101 strain demonstrated significantly higher GUS expression (1.7-fold) than the other two strains (Fig. 4a), and it therefore should be the optimal choice for *C. sativa*. Ti-plasmids are classified into several types based on the opines; EHA105, LBA4404 and GV3101 are grouped into the succinamopine, octopine and nopaline types, respectively^25^. Since GV3101 showed high transient GUS expression, octopine-type strains might have better compatibility with *C. sativa*, and other *Agrobacterium* strains belonging to this type, such as C58C1, GV3100, GV3850, A136, and EHA 101, should be tested for hemp agroinfiltration.

*C. sativa* is a dioecious plant, but to overcome the unsynchronized maturity of male and female plants and the problem with mechanized harvesting, genetically monoecious diploid (2n = 20) hemp cultivars have been developed^31^. The dioecious cultivar CRS-1 exhibited significantly higher relative GUS expression than seven other varieties. Among the five monoecious cultivars, Santhica 27 presented the highest relative GUS expression, and therefore this cultivar will be used to optimize the agroinfiltration protocol for future studies due to the agronomical advantages of monoecious cultivars.

Interestingly, this optimized agroinfiltration protocol was applied to various hemp tissue/organs, including hemp female flowers, and resulted in high GUS and GFP expression (Figs. 5 and 6).

Recently, the PDS gene has been used to examine transient gene silencing systems in both monocots and dicots^32,33^. A mutant of this gene, which is a single copy gene in most plants, induces an albino phenotype and is easily detected through visual observations due to the reduction of chlorophyll content^34^. Transient expression of the *PDS* silencing vector decreased the PDS mRNA level to 20% compared with that of the mock treatment and led to an albino phenotype in the leaves, male flowers and female flowers (Fig. 7). These results revealed that the optimized hemp agroinfiltration system reported in this study was effective for gene silencing via the transient expression of hairpin RNA.

In conclusion, we report an efficient protocol for agroinfiltration of various hemp tissues, including female flowers that contain glandular trichomes where hemp phytochemicals are produced and accumulated at high concentrations. This method is therefore a promising technique for functional genomics of hemp genes, particularly for the synthesis of cannabinoids, terpenoids, flavonoids and alkaloids. Since there are several reports regarding the production of proteins related to vaccines, pigmentation, photosynthesis and pharmaceutical components via agroinfiltration^13,35,36^, this technique can also play an important role in the metabolic engineering of hemp, for example by overexpression of the *CBDAS* gene and/or silencing of the THCAS gene.

## Materials and Methods

### Plant materials and growth conditions

The study was performed and the hemp plants were grown in accordance with the approved guidelines for Industrial Hemp provided by the Pennsylvania Department of Agriculture - Bureau of Plant Industry under regulated permits IH-16-P-2017 and IH-17-P-2017.

Six monoecious industrial hemp cultivars: Fedora 17, Felina 32, Ferimon, Futura 75, Santhica 27 and USO31 were provided by the Pennsylvania Department of Agriculture, and two dioecious cultivars: CRS-1 and CFX-2 were purchased by Hemp Genetics International (Saskatoon, SK, Canada). They were grown in the greenhouse at 21 °C with a 14-hour light photoperiod at 25–40 μEm^−2^s^−1^.

For *in vitro* growth, the hemp seeds were sterilized and transferred to germination media consisting of Murashige and Skoog (1962)^37^ (MS) with vitamins supplemented with 2% sucrose and 0.8% agar. The seeds were placed under fluorescent light with a 16/8-hour cycle for one week to allow germination and growth prior to *Agrobacterium* inoculation.

### Effect of plasmid, *Agrobacterium* strains and culture conditions

For overexpression, a pEarleyGate 101 vector (Invitrogen, Carlsbad, CA, USA) harboring the *uidA* gene and *eGFP* gene under the control of a cauliflower mosaic virus (CaMV) *35 S* promoter and *OCS* terminator was used for GUS staining and GFP fluorescence assays (Fig. 1a,b). An empty pEarleyGate 101 vector was used as a negative control (Fig. 1c).

The pEarleyGate 101-*uidA* was transformed into *A. tumefaciens* GV3101, LBA4404 and EHA105 strains^14,15^ and the pEarleyGate 101-*eGFP* vector was transformed into a GV3101 strain. For gene silencing, the hairpin RNA-expressing RNAi construct was prepared by inserting sense and antisense partial *CsPDS* fragments into the pK7GWIWG2 gateway vector (Invitrogen; Fig. 1d). A 200 bp *CsPDS* partial sequence (*PDS* 601–800) was amplified in the sense orientation from a cDNA library that was prepared using total RNA from hemp leaves. For cloning of the gene fragments into the entry vector Gateway pDONR/Zeo (Thermo Fisher Scientific, Waltham, MA, USA), primers were synthesized to include the recombination sequences *attB1* and *attB2*. Targeted gene fragments in the entry clones were then transferred to the binary silencing vector pK7GWIWG2(I)^38^ to form the hairpin RNA, which is under the control of a CaMV 35 S promotor and a CaMV *35* S terminator (Fig. 1e). The construct was evaluated by sequencing and subsequently transformed into *A. tumefaciens* GV3101 cells. The full-length *CsPDS* (PK24508) sequence information was obtained from the hemp genome gateway browser (http://genome.ccbr.utoronto.ca/cgi-bin/hgGateway).

A single colony of recombinant *Agrobacterium* was inoculated and grown in LB buffer. The culture was then harvested and centrifuged at 4,000 g for 10 minutes and resuspended to an OD_600_ of 0.5 in agroinfiltration media containing 10 mM MES, 1x MS, 2% glucose, and 200 µM acetosyringone at a pH 5.6. The chemical additives including Silwett L-77 (Lehle seeds, Round Rock, USA), Pluronic F-68 (Gibco, NY, USA), L-Ascorbic acid (Sigma-Aldrich, St Louis, MO, USA) and polyvinylpyrrolidone (PVP) (Sigma-Aldrich) were added before vacuum infiltration.

### Vacuum infiltration

Hemp tissues/organs were excised from the plants two months after seed germination, placed on a 100 ×15 mm Petri dish filled with *Agrobacterium* solution and placed in a vacuum chamber. The vacuum pump was turned on to decrease the pressure, and the agroinfiltration time was calculated after the vacuum reached 80 mbar. Successful infiltration required bubbles to be flowing up from the hemp tissues/cultures. After 5–15 minutes at low pressure, the release valve was opened slowly to allow entrance of *Agrobacterium* into the interstitial spaces of the plant tissues. The plant tissues were then washed with distilled water, transferred onto a moist filter paper in a Petri dish and placed in a growth room at 21 °C. Sonication was carried out using a 22.5 kHz XL2000 Qsonica sonicator (Qsonica LLC, Newtown, CT, USA).

### Analysis of transient GUS expression

Four days after agroinfiltration, the plants were analyzed by histochemical staining for GUS activity in the hemp tissues/organs as described by Bakshi *et al*. (2011) with some modifications^24^. The infiltrated plant tissues/organs were washed for 30 minutes in ice-cold 100 mM phosphate buffer (pH 7.0) and then vacuum infiltrated in 5-bromo-4-chloro-3-indolyl-beta-D-glucuronide (X-Glu) substrate solution [0.0005% X-Glu (w/v), 100 mM phosphate buffer pH 7.0, 1 mM potassium ferrocyanide, 1 mM potassium ferricyanide, 0.05% Triton X-100 (w/v)]. The GUS-stained tissues/organs were then submerged in 95% ethanol overnight and washed with sterilized water for decolorization.

The GUS-stained hemp tissues/organs were placed in a Petri dish and observed using a Nikon SMZ1500 microscope (Nikon, Tokyo, Japan) at 2x magnification with Imaging Software NIS Elements F Package (Nikon) and equipped with a DS-Ril1 camera (Nikon). The relative GUS expression was calculated with 30 pollen sacs per treatment to obtain the total expression value (stained area x color intensity) with ImageJ (Rasband 1997–2011) as previously reported^15^. Photos of the strongest GUS-stained regions in the pollen sacs were taken using bright field illumination. Prior to quantification, the color mode of the light microscope images was converted from RGB to HSB using ImageJ. A rectangular region of interest (ROI) was defined by using the “Rectangular selection” drawing tool from the ImageJ toolbar. The intensity of the GUS staining was measured in the saturation channel. The mean gray values were measured and compared among different treatments. All analyzed tissues were thoroughly cleared to correlate the color information with the degree of blue staining. In addition, the number of stained pollen sacs was counted from 10 pollen sacs to calculate the percentage of GUS staining. This calculation included three repetitions. Statistical analysis was performed using a 1-way ANOVA with Tukey’s multiple comparison test (a = 0.05).

### Image collection and analysis of GFP fluorescence

Four days after vacuum agroinfiltration, the hemp tissues/organs were subject to GFP fluorescence observation using a Nikon SMZ1500 microscope with Imaging Software NIS Elements F Package (Nikon) equipped with a DS-Ril1 camera (Nikon), an Intensilight C-HGFI Precentered Fiber Illuminator (Nikon) and a GFP2 filter set (Ex. 480 ± 40 nm; Em. 510 nm LP).

### Quantitative real-time PCR (qPCR) analysis of the agroinfiltrated hemp plants

Real-time PCR was performed with 5 μL of SYBR Select Master Mix (Applied Biosystems, Waltham, MA, USA) in a 10 μL total reaction mixture containing 400 nM of the gene-specific primers and 1 μL of cDNA. The following thermal program was used: 95 °C for 10 m, 40 cycles at 95 °C for 10 seconds, and 60 °C for 1 minute carried out with a CFX-96 Touch Real-Time PCR Detection System (Bio-Rad, Hercules, CA, USA). The gene expression level was calculated from the cycle threshold according to the 2^−∆∆Ct^ method^39^. Statistical analysis was performed using a paired t-test (a = 0.05). The PDS gene primers were designed to amplify a region upstream of the siRNA sequence (*PDS* 466–555). The *F-box* gene was used as an internal reference for hemp, and three independent biological replications were conducted. All primer sequences are listed in Supplemental Table S1.

## Supplementary information

Supplementary Information.
