# Supplementary material for: Establishment and optimization of a hemp (Cannabis sativa L.) agroinfiltration system for gene expression and silencing studies
Source: Sci Rep. 2020 Feb 26;10:3504. doi: 10.1038/s41598-020-60323-9 (PMC7044430; doi:10.1038/s41598-020-60323-9)
Supplement: Supplementary file 1 — Supplementary Information. [file 41598_2020_60323_MOESM1_ESM.pdf]

Additional Supplementary Information

**Supplementary Table S1. Primer sequences used in this study.**	
**Genes**	**Primer sequence (5'-3')**
CsPDS	F: CTTGGCAGATGCAGGTCATA<br>R: TCACCGTCATCATCTTTCCA
CsF-box	F: TATCGGCGGAGAGATTTGAG<br>R: TAAGCCCTTCCCTTGATTCC
uidA Fw	F: GGTGCACGGGAATATTTCGC<br>R: ATAACGGTTCAGGCACAGCA
pEarleyGate 101-uidA	F: GGGGACAAGTTTGTACAAAAAAGCAGG<br>CTTCACAATGTTACGTCCTGTAGAAAC<br>R: GGGGACCACTTTGTACAAGAAAGCTGG<br>GTCTTCATTGTTTGCCTCCCTGCTGCG
pK7GWIWG2(I)-CsPDS	F: GGGGACAAGTTTGTACAAAAAAGCA<br>GGCTTCACACTTTGGAGCTTATCCCAA<br>R: GGGGACCACTTTGTACAAGAAAGCTG<br>GGTCTTCACCCAGGTCAGCATCTCATTG
